# Modelling vulnerability: qualitative study of the Balint process for medical students

**DOI:** 10.1186/s12909-022-03508-2

**Published:** 2022-06-07

**Authors:** Lillian Ng, Chris Seu, Sarah Cullum

**Affiliations:** 1 The University of Auckland, Auckland, New Zealand; 2 Counties Manukau Health, Auckland, New Zealand

**Keywords:** Balint, Medical students, Medical education, Psychiatry, Doctor-patient relationship, Emotional processing, Reflection, Self-integration

## Abstract

**Background:**

Health professionals in training may be overwhelmed by the emotional dimensions of their work. Balint groups have been proposed as an intervention to support students to process emotional aspects their encounters with patients. The aim of this study was to explore medical students’ emotional experiences of a Balint group during their psychiatry attachment.

**Methods:**

Medical students completing a six week clinical attachment in psychiatry took part in weekly Balint group sessions. Five sessions were audio-recorded, transcribed and coded by members of the research team and an independent researcher co-coded all transcripts. Themes were discussed and refined over three rounds of coding.

**Results:**

Four themes were identified: the Balint process as a container to enable vulnerability; becoming attuned to clinical and professional encounters; an evolving sense of identity within the medical hierarchy; and, the need for self-preservation to retain empathy for others.

**Conclusion:**

The Balint structure provides medical students with a secure and emotionally resonant experience. Medical students’ engagement with the Balint process, even over a relatively short time period, teaches them how to reflect on difficult emotions associated with clinical encounters. Early exposure during a psychiatry placement may benefit students in terms of understanding relationship dynamics and the need for self-integration and lifelong reflection over the course of their medical career.

## Introduction

At the centre of medicine is the relationship between a doctor and their patient [1]. Each has a significant influence on the other, an effect described as so powerful in terms of human connection that doctor and patient cannot be considered separately. The tradition of Balint is well established in primary care and increasingly used by other health professionals. Balint groups assist clinicians to explore clinical encounters and examine their personal reactions [2].

Balint groups are of value during general medical training in developing students’ empathy and communication skills, particularly in family medicine [3–5]. A systematic review investigating Balint groups in undergraduate training found that medical students developed empathy and communication skills [6]. The Balint process may foster basic psychological competency and an understanding of case dynamics [7]. In psychiatry, we teach students about affect as an essential dimension of the mental state examination. Affect is a term that encompasses mood, feeling, attitudes and preferences [8]. Despite evidence that focusing on emotional responses to patient care improves clinical care, relationships and work satisfaction, medical programmes may have a less organised approach to promoting personal awareness. Balint work engenders self-awareness as group members explore different aspects of their professional identity, express difficult emotions and gain others’ perspectives [9]. The emphasis on self-reflection within the group process may shift members’ attitudes toward patients, even within a relatively short period of time [10]. Patients benefit as clinicians apply insights to their own practice and interact with patients differently [11].

At our medical school, students commence clinical work in year 4 of their undergraduate training. In year 5, they undertake a clinical psychiatry rotation (six weeks) and in year 6 they complete a further psychiatry rotation (four weeks). Our teaching combines clinical time with patients, didactic lectures and interactive small group work. During teaching sessions we observed students conversing about challenging patient encounters. Students reported debriefing with senior colleagues, peers, family members and friends. Many articulated a desire to process their difficult feelings within a safe environment. For this reason we introduced the Balint group process to year 5 medical students and asked for their feedback on the experience.

We were curious about the ways that medical students process difficult feelings in working with patients. We hypothesised that Balint groups, as an intervention, could support students to attend to and process emotional dimensions of their encounters with patients. We considered a psychiatric placement an apt time to study Balint groups during clinical training.

The objective of our study was to explore the emotional experiences of year 5 medical students who participated in a Balint group during a six week psychiatry rotation and to examine potential benefits from their engagement with the process. Case material was presented using a structured process led by a facilitator. As part of the process, the presenter stepped back from the group and observed them processing the encounter. The facilitator drew attention to emotional aspects of the clinician-patient interaction and encouraged members of the group to hypothesise about emotional aspects of the encounter as opposed to dwelling on clinical concerns or problem solving. Towards the end of the session, the presenter was invited to re-join the group and participate in the discussion.

## Methods

### Study design and sample

The authors are academic and clinical tutors in the specialty of psychiatry. Two authors are psychiatrists with a formal teaching role and one author is a psychiatric trainee. Our clinical work is located in a unique location, an ethnically diverse catchment area [Counties Manukau Health]. We chose a qualitative methodology to explore the use of Balint and potential benefits of engaging with the process. Our sample was a group of year 5 medical students who completed a six week rotation in psychiatry during the 2019 academic year. Following ethics approval, we asked a university coordinator to email a letter of invitation and participant information sheet to the year 5 medical students at our teaching site. They took part in a weekly Balint group session over six consecutive weeks. Students were given the opportunity to ask questions about the study on the first day of orientation. Signed consent forms were collected prior to the first session.

The second author, a senior psychiatry trainee, facilitated the Balint groups. The confidential nature of discussion was emphasised as was the importance of student wellbeing and sources of further emotional support. Students were given an opportunity to present a case they experienced during the rotation, or at another point in their training, particularly one that evoked a strong personal reaction. As part of the process, the group were invited to ask questions of the presenter to clarify aspects of the case. The presenter subsequently moved their seat away from the group circle and observed in silence, while the rest of the group reflected on the case. At the 45 min mark, the presenter was invited to return to the group and reflect on the discussion. Each session was of one hour duration and recorded using a digital recorder. The final session focused on evaluating the experience of the Balint process, using a semi-structured questionnaire.

### Data analysis

The audiotaped recordings were transcribed and identifying content was removed. Participants were allocated a numerical code, and names were de-identified in the transcripts. The recordings and transcripts were stored on a password-protected university server. Following transcription, the audiotaped recordings were deleted. The transcripts were analysed using the thematic analysis as described by Braun and Clarke [12]. The research team independently read and coded each transcripts. Reflections on the data were documented as part of the audit trail. The researchers compared their findings together and constructed an initial coding framework. A researcher with expertise in qualitative methodology was employed to independently analyse the six transcripts. The authors met with the independent researcher; discrepancies were robustly discussed and a consensus was drawn on key themes.

## Results

Six Year 5 students participated in the study. We identified four themes over the course of six sessions and provide illustrative quotations: the Balint process as a container to enable vulnerability; becoming attuned to clinical and professional encounters; an evolving sense of identity within the medical hierarchy; and, the need for self-preservation to retain empathy for others.

### The Balint process provides a ‘container’ to enable vulnerability

The Balint process was an opportunity to revisit a case and use the group process to resolve latent feelings. Participants identified challenges in caring for patients, power imbalances within the medical hierarchy and dealing with intense clinical encounters. The structure of the Balint group provided participants with a unique opportunity for reflection:

I found it quite useful reflective practice. In terms of talking about the cases and seeing how other people felt and think.

The participants described the supportive environment as counterculture to attitudes portrayed by their senior colleagues:

There’s still definitely that attitude in medicine. That you should just toughen up.

If you toughen up it suggests that you should be devoid of feeling, which is obviously not possible.

Many acknowledged the Balint process provided a sense of safety and deepened trust within the group. They reported being able to discuss their inexperience and identified with what their peers had faced:

Can I just be the first to say that that’s very terrifying to be put in that situation.

I would have felt very out of depth and not sure.

Some spoke of trying to maintain a professional veneer by shrugging off feelings:

Like most things in medical school, you just get immune... you see hundreds of patients, and you can’t [feel], you just have to put that aside and move on.

Most perceived an increased stress, related to the demands of medicine. The group enabled expression of their raw feelings and vulnerability:

I find medicine quite hard because I’m worrying a lot more about things…it’s all right, I didn’t mean to get upset.

It’s really hard when it’s someone like you. I’m gonna start crying.

The participants reflected on feeling less alone after hearing peers speak about their discomfort. They were insightful about their growth in learning by hearing others’ perspectives.

It made me realize what I thought at the time and a lot of biases, mistakes, things that weren’t so good…I can see it in a separate light now talking about it. I feel more aware now.

### Attunement to clinical encounters and professional dynamics

Participants described becoming aware of complex dynamics between doctors and patients and within the clinical environment. They realized they were in a rare position as part of a team within the hospital milieu:

As a student, you have a unique perspective on the situation….you’re kind of an observer, seeing everything happening. You’re seeing the family, you’re seeing the doctors, you’re on the ward round. You’ve got this unique view that no one else has.

Some observed situations of direct conflict unfolding between patients and staff. They lacked agency to intervene and felt unsure about what they should say or do:

I see the family member, angry at the staff … the staff frustrated, fed up and angry at the family member. I’m sitting there on the fence as an observer, watching the situation bounce back and forth.

Some participants reported their inclination to be empathetic was discouraged by senior colleagues:

Some of the staff made me question whether my empathy was misguided because they tell me that I’m being naïve. And that I’m young.

Students reported being invested in patient outcomes and feeling ambivalent when they were not able to follow up patients in the long-term. Some participants identified strongly with patients’ suffering:

She was 26, female, similar build to me, she was wearing an engagement ring and it was very confronting, given that [she] was quite similar to my demographic. It was quite overwhelming... sorry I’m getting upset, I don’t mean to be, I could see myself in that situation, that was a bit hard.

### An evolving sense of identity within the medical hierarchy

The participants discussed the struggles of finding their voice and the confidence to speak up for patients. They also articulated a sense of powerlessness within the medical hierarchy as they reflected on salient clinical experiences. Many articulated ‘feelings of inadequacy:’

I don’t feel qualified to make the decision that this is an emergency. Like I don’t know, I haven’t seen enough to be sure. A mum comes out with her baby, “Quick, help. He’s not breathing.” I was like, “Oh my God.” So I ran there and…. I was trying to figure out what to do.

Many described feeling hesitant when they needed to respond to clinical situations with some urgency:

I wasn’t familiar with the ward set up, I couldn’t see the emergency button and I didn’t know whether I should ring…whether as a student I was allowed to do it… thankfully the nurse came in and I stepped away... but yeah, I felt very very unsure, I don’t know what I would’ve done if the nurse hadn’t come in.

They also described feeling ambivalent and anxious about determining whether to seek help and in locating assistance:

I was like, “Okay, do I need to get more help now, what do I need to do at this point in time?” I didn’t know who his physician was and I can’t really go and find them because they were on ward rounds.

Some deferred to their senior colleagues as they struggled to speak up or advocate for patients:

I felt relieved that [the registrar] took control of the situation, but I also felt guilty.

The participants discussed the difficulties of finding their voice and the confidence to speak up for patients. They also articulated working out their identity as doctors and determining actions that were appropriate for their stage of training. 

### The need for self-preservation to retain empathy for others

The participants reported that the Balint process gave them an opportunity to revisit and discuss cases that had ‘been shelved away.’ They acknowledged that talking about what upset them made them feel less burdened:

The first case I presented I still think about but it doesn’t trouble me like it used to. It’s helpful to know that other people had similar feelings.

The participants were reassured that others had articulated similar feelings and reactions. They highlighted self-awareness and learning from the group as a way to counter stress in the long term:

I’m quite scared that once pressure starts to mount and I start to have some responsibility that the irritable edge will come out towards my colleagues, sometimes my patients too. Being aware of it is a good first step.

Taking part in the Balint group highlighted awareness of the need for ‘self-preservation’ and the high cost of becoming a member of the caring profession:

Empathy takes up a lot of energy…you’ve got to make sure that you take time to self-care so you can be empathetic…I find it takes quite an emotional toll. If I don’t have time for myself or around people that I love, it’s harder. I hope that after medical school that will get easier.

They were also mindful of feeling squeezed as part of a ‘system’ that did not necessarily model good self-care:

It’s very hard to be staying empathetic in a system that doesn’t really allow us to preserve our empathy. That isn’t empathetic towards us.

Some participants emphasised that they did not want to ‘toughen up’ and lose their feelings or empathy for others:

I don’t know whether that’s just because I’m a student and I’m fresh and I’ve got a lot of energy to give. It’s hard when I get very emotional but it’s also really awful to not have any feelings.

When participants felt distressed by their clinical experiences they often thought about them after work. They were aware of the importance of having a way to debrief rather than taking their concerns home:

I just couldn’t stop thinking about it all night. I know it didn’t help I got home at midnight… you don’t really have the chance to wind down.

Participants found it helpful to discuss clinical challenges at the time they had occurred, rather than weeks or months later. They were appreciative of insightful colleagues who sensed they were struggling or affected by clinical experiences. They valued discussing cases at the Balint group and were surprised by the intensity of feeling that the Balint process evoked.

## Discussion

Our findings suggest that the Balint process benefits medical students by providing a structure that enables an emotionally enriching experience. There was sufficient trust within the group for students to be vulnerable and “unpack” feelings about clinical encounters. This led to wider discussions about relationships with patients, colleagues and the hospital system. The students’ reflections made them feel less alone as they realized their peers had similar experiences. The data analysis reflected students’ increasing emotional awareness, trust of other group members and engagement and the Balint process.

We agree that Balint groups can powerfully contribute to medical education if they are part of the medical curriculum [11]. As clinicians working in psychiatry, we value the reflective process of noticing and processing emotions. Medical students experience distressing feelings when they are confronted by illness and death [13, 14]. In Balint work, creating an environment of psychological safety for students is ultimately important to enable them to share difficult feelings. Facilitators need to be sufficiently skilled and sensitive in guiding and containing the group process to engender reflective functioning within the group [15]. In the course of training, students gain proficiency in diagnosing and treating patients albeit with growing awareness that skill acquisition is only one dimension of clinical work [16]. Reflection is an essential part of forming a professional identity [17] and there may be little emphasis on the emotional labour that leaves health professionals drained and losing their empathy [18]. In our study, students opted to attend which may have self-selected those more inclined to introspection. Balint groups may offer receptive members a better capacity to tolerate uncertainty and an increased ability to empathize with patients [13]. Our students utilised the security of the group [19, 20] to connect to each other [21] and focus on their emotional responses [7].

The final theme of understanding the need for self-preservation in order to retain empathy may have important implications for future practice. Balint groups may have longer term benefits as students recognise the importance of protecting and caring for themselves in order to sustain their empathy for others [9]. Our students came to realize that they needed to fortify themselves emotionally to deal with the complexity and uncertainty of clinical practice [16]. The Balint process may address concerns about resilience, burnout and compassion fatigue [6]. Balint work may benefit medical students differently from clinicians who have ongoing patient relationships and responsibilities [11]. Our students discussed their feelings about patients but also focused on concerns related to professional identity, role models and relationships with other health professionals [3]. They were painfully aware of their position within the medical hierarchy as they observed conflicts and tensions [17]. During the course of six weeks, we discerned professional growth in students with self-integration as they were more adept at identifying their feelings and the impact these might have on relationships with patients and staff.

Balint groups provide a structure and security to model the healthy expression of emotions. Giving medical students early experience of the Balint process is counter to the view that doctors should detach and toughen up. There is a cost to avoiding speaking about emotions and we do so at our peril [13]. Students bear witness to pain and suffering, and this may make them feel uneasy and distressed. In psychiatry, we can role model how to lean into our emotions as an act of self-compassion. As students understand more about relational dynamics related to difficult encounters they may come to view their patients more holistically. The benefits of Balint for medical students have led us to continue facilitating them during the Year 5 psychiatry rotation, even during COVID-19 related disruptions, where we utilised videoconferencing to continue the groups.

## Strengths and limitations

The capture of rich data was facilitated by the second author, who led the Balint groups over a six week period and engendered a high degree of trust within the group. The rapport formed in the group allowed participants to freely express meaningful emotions and form connections with one another. This contributed to a rich data collection. A further strength was the independent coding of data and robust discussion of themes. Just one group of students was included in this study which may limit transferability of themes. The data analysis may be limited by the coders’ integrating the participants’ experiences in the Balint group process and their evaluation of it. As such, active aspects of learning and reflection are integrated.

## Conclusion

The Balint structure provides medical students with an emotionally resonant experience. The engagement of medical students with the Balint process, even over a relatively brief period of six weeks, teaches them how to reflect on and work through difficult emotions associated with clinical encounters. Early exposure during a psychiatry placement may have benefits in terms of becoming attuned to the dynamics of the doctor-patient relationship, self-awareness, self-integration and lifelong reflection.

## Data Availability

The datasets generated and/or analysed during the current study are not publicly available due to the confidential nature of patient case material included in group discussions but are available from the corresponding author on reasonable request.
